# Trauma Without Borders: The Necessity for School-Based Interventions in Treating Unaccompanied Refugee Minors

**DOI:** 10.1007/s10560-018-0552-6

**Published:** 2018-05-29

**Authors:** Diana Franco

**Affiliations:** 1 New York University Silver School of Social Work, 92-31 57th Avenue, Apt. 4A, Elmhurst, NY 11373 USA; 2 New York University Silver School of Social Work, New York, NY 10003 USA

**Keywords:** Immigration, Minors, Trauma, Unaccompanied refugees, Schools

## Abstract

This article explores migration trauma among Mexican and Central American unaccompanied refugee minors (URM) with the purpose of developing an understanding of migration as a tripartite process consisting of: pre-migration exposure to traumatic stressors, in-journey stressors, and post-migration stressors. The migration experience of these youth may be subjectively different depending on a wide range of factors. The complexities of migration are explored as a traumatic, tripartite process. These three salient components of migration may act as precursors, often resulting in psychological sequelae such as: post-traumatic stress disorder (PTSD), anxiety, and depression. Of all migrant groups, URM are more likely to develop psychiatric symptoms. Trauma-Focused Cognitive Behavioral Therapy (TF-CBT), Cognitive Behavioral Intervention for Trauma in Schools (CBITS), and Mental Health for Immigrants Program (MHIP) are among the most effective interventions in the treatment of PTSD, anxiety, and depression in refugee minors. Social workers in schools are in unique positions to provide mental health services to URM. A case example illustrating a cultural adaptation of TF-CBT in an urban public high school is included. Clinical implications of culturally responsive and trauma-informed treatment of URM in schools will be discussed. Additionally, this article will emphasize the importance of bridging the gap between research and culturally responsive, trauma-informed interventions for URM in schools.

The literature pertaining to the migration process of unaccompanied refugee minors (URM) from Mexico and Central America, explores the tripartite process, consisting of: (1) pre-migration exposure to traumatic stressors, (2) in-journey stressors, and (3) post-migration stressors, as adverse events, when approached through a trauma-informed perspective (Fazel & Stein, 2002; Kirmayer et al., 2011; Perez-Foster, 2001; Pine & Drachman, 2005; Sullivan & Simonson, 2016). Although most of the literature acknowledges the connection between migration and trauma, an in-depth discussion on migration trauma as a tripartite process and as a clinical framework for assessment and treatment is inconsistent and often missing. When compared to other migrants, URM are at a higher risk of developing post-traumatic stress disorder (PTSD) and other psychological sequelae such as depression and anxiety as a result of forced exile and exposure to traumatic events before, during, and after migration (Adelman & Taylor, 2015; Fortuna, Porche, & Alegria, 2008; Rasmussen, Crager, Baser, Chu, & Gany, 2012; Smid, Lensvelt-Mulders, Knipscheer, Gersons, & Kleber, 2011; Unterhitzenberger et al., 2015; Yearwood, Crawford, Kelly, & Moreno, 2007). Therefore, the unique needs of URM call for awareness and action by school-based social workers and educators in tailoring services that meet their complex needs after migration.

Immigrant minors are youth under the age of 18, who migrate to the United States from their countries of origin because of varied, complex dynamics known in the literature as *push and pull* factors (Kandel et al., 2014; Lee, 1966; Meyer, Margesson, Ribando Seelke, & Taft-Morales, 2016). *Push factors* are described as forces that cause migrants to flee their countries and/or play into the decision-making process to migrate to the United States. Push factors originate in the migrants’ native country, for instance war, poverty, and persecution (Lee, 1966). *Pull factors* are influences that originate in the resettlement country, such as reunification with family, that urge URM to migrate specifically to the US (Kandel et al., 2014; Meyer et al., 2016). Although migrant groups may differ in levels of exposure to traumatic events (Pumariega, Rothe, & Pumariega, 2005), pre-migration stressors, “in-journey” trauma-exposure, and post-migration stressors, are elements in the composition of migration trauma that contribute salient information to the youth’s psychosocial history (Kirmayer et al., 2011). Overall, push and pull factors will continue to influence the arrival of URM in the United States in years to come.

In the US, schools provide most mental health services to minors (Bal & Perzigian, 2013; Beehler, Birman, & Campbell, 2012; Burns et al., 1995; Hoagwood & Erwin, 1997; Kataoka et al., 2003; Stein et al., 2002; Sullivan & Simonson, 2016). Overall, social work services in schools contribute to the reduction in emotional distress and academic challenges experienced by URM (Dods, 2015). Schools are situated to shorten the gap in service delivery for undocumented children and families who would not otherwise have access to these services as a result of transportation barriers, stigma, and lack of health insurance (Stein et al., 2002; Sullivan & Simonson, 2016). Although the literature (Green, 2003; Krogstad, Barrera, & Lopez, 2014) emphasizes an increasingly steady influx of unaccompanied minors migrating to the US (Pierce, 2015), there is a dearth of empirical research (Greenman & Hall, 2013; Rasmussen et al., 2012; Yearwood et al., 2007) pertaining to trauma-informed mental health services and culturally responsive treatment for URM in schools.

The role of pre-migration, in-journey, and post-migration stressors will be explored as precursors for PTSD and other psychiatric disorders in URM. The relationship between trauma-related, psychological sequelae and their effect on learning and academic achievement will be discussed. The necessity for evidence-based interventions such as Trauma-Focused Cognitive Behavioral Therapy (TF-CBT), Cognitive Behavioral Intervention for Trauma in Schools (CBITS), and Mental Health for Immigrants Program (MHIP) will be explored. Culturally responsive modifications will be discussed through a tripartite process of migration framework to emphasize the importance of making accurate assessments and establishing appropriate supports in schools. The need for longitudinal, multimodal, and participatory action research (Stein et al., 2002) to address the gaps between research and culturally response practice with URM will be emphasized.

## Literature Review

### Mexican and Central American Unaccompanied Refugee Minors: Who are They?

According to the 2016 United Nations (UN) Global Trend Report, of the world’s 65.3 million people who are forcibly displaced, more than half are young people under the age of 18 (UNHCR, 2016). In the United States, almost 40% of new refugees are minors; although the literature posits that these statistics are difficult to monitor because URM often lack appropriate identification (Sullivan & Simonson, 2016). According to a report by U.S. Customs and Border Protection (2018), between October 2016 and September 2017 41,435 unaccompanied youth had been apprehended at the US Southwest border. In 2017, 17% of these unaccompanied youth were between 0 and 12 years of age, 13% between 13 and 14 years of age, 37% between 15 and 16 years of age, and 32% were 17 years old (Office of Refugee Resettlement, 2018). The Office of Refugee Resettlement (2018) notes that in 2017, 68% of the youth apprehended were male and 32% were female. After the surge of unaccompanied minors in 2014, there continues to be a steady, yet increasing influx in rates of immigrant youth (Pierce, 2015) and URM to the US. This proportion is likely to grow significantly by 2020 (Yearwood et al., 2007).

URM are youth under the age of 18 who flee their countries without an adult companion and do not face the option of returning to their homeland (Fong, 2007). The UN defines URM as youth “who are separated from both parents and are not being cared for by an adult who, by law or custom, is responsible to do so” (Unterhitzenberger et al., 2015, p. 1; UNHCR, 2014). Additionally, fear of persecution for reasons of race, religion, social or political affiliation, nationality, and lack of protection from country of origin, may result in an inability or unwillingness for URM to return (UNHCR, 1951). Thus, as a migrant group URM are distinct because of the persecution and displacement they experience when compared to other migrant groups (Sullivan & Simonson, 2016).

The term *immigrant minors* is usually discussed amorphously in the literature; resulting in broad discussions and generalizations about a non-homogenous population (Bal & Perzigian, 2013). Immigrant minors are characterized by diversity that encompasses differences from migratory status, type of migration route utilized, and whether the youngsters migrated unaccompanied by an adult (Fong, 2007). In addition, there are other noted distinctions between voluntary immigrant minors and URM, such as the latter usually being undocumented. Voluntary immigrant minors often embark on migration from their countries of origin, to join other family members in the US and seek better life opportunities (Adelman & Taylor, 2015; Fong, 2007). Push factors force URM to flee their countries of origin because of political unrest, civil war, gang violence, or persecution (Adelman & Taylor, 2015; Fong, 2007). Both groups of migrants leave a great part of their cultures and loved ones behind and may struggle with traumatic grief, adjustment, and racism upon arrival to the US (Adelman & Taylor, 2015; Fong, 2007).

Krogstad, Barrera, and Lopez (2014) note that URM from Central America and Mexico are the fastest growing group of immigrants in the United States. From October 1, 2012 to May 31, 2014, 46,932 unaccompanied children were taken into custody by U.S. Customs and Border Protection (Krogstad et al., 2014). Other research (Pierce, 2015) indicates that 102,327 unaccompanied children were apprehended at the US-Mexico border from the start of 2014 (fiscal year) through August 31, 2015. Most URM come to the US from Mexico and the *Northern Triangle*, which refers to El Salvador, Guatemala, and Honduras—countries plagued by civil wars, gang violence, and severe poverty (Ciaccia & John, 2016; Pierce, 2015; Stein et al., 2002; Zatz & Rodriguez, 2015). Between 2014 through August 31, 2015, a total of 76,572 URM from the Northern Triangle were apprehended at the US-Mexico border (Pierce, 2015). Furthermore, Central America has the highest homicide rates per country, especially among males ages 15–29 (Ciaccia & John, 2016). In 2012, The Women’s Refugee Commission interviewed 151 URM, 77% of which stated that violence in their home countries was their primary reason for migrating (Ciaccia & John, 2016). Of the minors interviewed, most reported that if they had the opportunity to “repeat the journey to the United States,” they would (Ciaccia & John, 2016, p. 1).

For the most part, Mexican unaccompanied minors are deported immediately (Pierce, 2015) and few Mexican migrants have been granted asylee immigration status in the US (Greenman & Hall, 2013). In contrast, migrants from the Northern Triangle are usually admitted to the US as refugees. If proved eligible, URM can change their legal status to asylee, “a protection granted to foreign nationals already in the United States or at the border who meet the international definition of a refugee” (American Immigration Council, 2017, para. 2; Greenman & Hall, 2013). It is important to note the differences between the designations *refugee* and *asylee*. Refugees and asylees must both meet the same legal definition of having a well-founded fear of persecution due to race, religion, nationality, or membership in a particular social group. Refugees receive legal permission to resettle in the U.S. before they arrive, whereas asylees must meet the definition of refugee and apply for asylum after they arrive in the U.S. (American Immigration Council, 2017).

Consequently, in immigration court, Central American URM, may contest deportation by requesting that they be granted formal relief, which grants immigration status of asylum or Special Immigrant Juvenile (SIJ) (Pierce, 2015). Migrants from Guatemala and El Salvador have been admitted to the US as refugees since the 1980s, following political unrest in these countries (Greenman & Hall, 2013). Honduran migrants have qualified for Temporary Protected Status (TPS), after hurricanes, floods, and other natural disasters have affected the area in the 1990s and early 2000s (Greenman & Hall, 2013).

## Migration as a Tripartite Process

### The Impact of Exposure to Pre-migration Adverse Events

Migration is a tripartite process, consisting of pre-migration, in-journey, and post-migration stressors, that may be explored through a trauma-informed perspective (Fazel & Stein, 2002; Perez-Foster, 2001; Sullivan & Simonson, 2016). Although each URM report unique migration stories, there are commonalities in these narratives that URM experience as a collective (Murray, Cohen, Ellis, & Mannarino, 2008). Pre-migration events may expose URM to a wide array of stressors that widely depend on their country of origin’s political and socioeconomic histories.

Overall, out of all emigrating groups, refugees of all ages are the only group whose exposure to significant pre-migration violence results in psychiatric symptoms (Yearwood et al., 2007). URM, as previously mentioned, are forced to flee from countries and regions plagued with civil unrest, violence, war, and other push factors. These push factors are believed to play a role in exposure to traumatic events prior to migration, increasing the youth’s susceptibility to developing PTSD (Smid et al., 2011). Due to the possibility that it may take longer for people with PTSD to seek help (Parsons & Ressler, 2013), it is important for service providers to understand pre-migration adverse effects through a trauma lens. Parsons and Ressler (2013) underscore “that exposure to trauma in the past, before the ‘index trauma’ associated with PTSD—particularly childhood trauma exposure—is of substantial importance” (p. 147).

Scholars (Sawyer & Márquez, 2017; Tello, Castellon, Aguilar, & Sawyer, 2017) emphasize that gang violence is one of the push actors which result in forced migration from Central America. URM participants in a thematic analysis by Tello et al. (2017) “discussed fleeing to escape gang violence and death” (p. 368). One participant in the study by Tello et al. (2017), reported being forced to flee Central America after losing her parents to gang violence and having been threatened by a local gang. Similar literature (Garsd, 2015) reports that a 15-year-old girl from El Salvador was shot to death by gang because she was selling tortillas in a rival gang’s territory. Garsd (2015) adds that a different 15-year-old girl was shot in the head because her boyfriend refused to join a gang. Similar gang-related violence is experienced in Guatemala and Honduras.

War, violence, living in unsettled refugee camps, and persecution are events that often go hand-in-hand with physical and emotional trauma (Adelman & Taylor, 2015; Fong, 2007). When compared to voluntary immigrant youth, URM were more likely to report a higher incidence of trauma related to war and were disproportionally impacted by it (Collier, 2015; Pumariega et al., 2005; Rasmussen et al., 2012). The countries that make up the Northern Triangle have had a long history of violence and civil unrest. El Salvador experienced a civil war throughout the 1980s and 1990s (Sawyer & Márquez, 2017). As a result, violence and persecution spilled into the neighborhoods in the form of executions and decapitations (Sawyer & Márquez, 2017). The military coup of 2009 in Honduras resulted in police corruption and unchecked crime against journalists, people who identify as LGBT, and peasants (Sawyer & Márquez, 2017, p. 70). A 36-year-long civil war in Guatemala, which began in 1960 and ended in 1996, has left a lasting impact on the country (PBS, 2011). Violence, intimidation, and organized crime continue to be a problem in Guatemala (PBS, 2011), forcing URM to flee their native lands.

Exposure to living in war-affected regions and violent environments has been known to affect the mental health of Latino migrants and refugees (Fong, 2007; Fortuna et al., 2008; Yearwood et al., 2007), resulting in PTSD and depression (Rasmussen et al., 2012). The literature (Barowsky & McIntyre, 2010; Fong, 2007; Fortuna et al., 2008; Rasmussen et al., 2012; Yearwood et al., 2007), shows that depression and PTSD have been noted in children and adolescent asylum seekers fleeing from civil conflict or war affected regions and that there is an average of 9 years, of pre-migration PTSD onset (Rasmussen et al., 2012). PTSD onset has been challenging to measure due to migration circumstances that make assessment difficult (Smid et al., 2011). Smid, Lensvelt-Mulders, Knipscheer, Gersons, and Kleber (2011) argue that late-onset PTSD in URM is closely associated with age of migration and lower education levels. This was noted between one to 2 years after resettlement (Smid et al., 2011).

URM have also reported being exposed to other forms of pre-migration violence not related to war, such as witnessing the violent death of loved ones, community violence, being threatened with a weapon, kidnapping, domestic violence, sexual and physical abuse (Fortuna et al., 2008; Rasmussen et al., 2012; Smid et al., 2011; Yearwood et al., 2007). These pre-migration traumatic events, coupled with severity of exposure, may predict future traumatic events and susceptibility to PTSD (Rasmussen et al., 2012; Smid et al., 2011). Other pre-migration stressors that may contribute to PTSD and other reported psychological sequelae include: the physical and emotional effects of poverty, malnourishment, physical and mental health issues, substance/drug abuse, educational achievement, teen pregnancy, grief, personal losses (home, family, friends, belongings), leaving countries of origin suddenly without saying good-bye to loved ones, and being unaccompanied (Fong, 2007; Smid et al., 2011; Yearwood et al., 2007).

### Migration “In-Journey” Stressors and Risk of PTSD and Other Psychological Sequelae

Previous literature (Rasmussen et al., 2012) underscores differences in migration histories between URM and voluntary immigrant youth. The most significant difference between these groups is the migration method used by URM to come to the US. URM from the Northern Triangle embark on their journey in many ways. The aid of a *coyote*, or human smuggler, is sought by families who can send money from the US to pay for the coyote’s services. Human smugglers, unlike human traffickers, transport people across international borders who voluntarily seek this service. Nonetheless, human smuggling is a crime (Vargas, 2014). The migration journey can be treacherous, exposing the URM to being assaulted, physically and sexually abused, and sustaining physical injuries. URM also face the risk of dehydration and malnourishment from long periods of traversing through arid terrains, while avoiding government officials.

Many URM take buses, vans, and other methods of transportation arranged by coyotes (Dominguez Villegas, 2014), as they find their way through Central America and Mexico. However, one method that has attracted attention from US and Mexican policy makers and human rights activists alike, is *La Bestia* (The Beast), freight trains that transport a variety of products to the US (Dominguez Villegas, 2014). Many URM who take La Bestia as their method of migration to the US are among the poorest of migrants, however, hitchhiking a ride on La Bestia offers a cheaper alternative to paying coyotes (Dominguez Villegas, 2014). Designed for cargo, La Bestia offers no passenger railcars, forcing URM who use this method to ride on top of the freight trains; exposing riders to serious risks (Dominguez Villegas, 2014).

URM aboard La Bestia are vulnerable to a multitude of dangers and traumatic stressors, such as the possibility of falling or being pushed off the freight train, amputation, and death (Dominguez Villegas, 2014). Moreover, URM aboard La Bestia are vulnerable to extortion, theft, or sexual assault by gangs who may force them to pay “protection” fees. These gangs have been reported to work with Mexican organized crime groups by threatening and bribing migrants in return for safe passage (Dominguez Villegas, 2014). In a study by Tello et al. (2017), some participants “reported being beaten and robbed in Mexico when their train would stop at various points” (p. 365). Train conductors, often part of the smuggling and extortion operations, demand bribes of families, women, and children (Dominguez Villegas, 2014).

Exposure to these extreme conditions, experiencing and/or witnessing death, abuse, and other forms of violence, increase susceptibility for the development of PTSD and depression (Yearwood et al., 2007). However, PTSD and other psychological sequelae may result from other losses not related to violence during the migration journey. Murray, Cohen, Ellis, and Mannarino (2008) assert that refugee populations, among the aforementioned stressors, are characterized by loss and traumatic grief. Grieving about deceased loved ones, sudden separation from family, friends, and country of origin may lead to feelings of resentment, anger, and guilt in URM (Murray et al., 2008). However, irrespective of the migration method used, most URM traveling through Central America and Mexico, may have already been impacted by violence, poverty, and war in their countries of origin. The exposure to these events only serves to exacerbate exposure to previous trauma.

### Post-migration Resettlement Stressors

#### The Icebox: US Detention Centers

The end of migration’s hazardous journey marks the beginning of the post-migration phase, which is characterized by a unique set of stressors. Upon crossing the Southern US border, URM are apprehended by US Customs and Border Protection (CBP). Consequently, a series of processes that determine the fate of URM in the US begin to unravel.

According to the William Wilberforce Trafficking Victims Protection Reauthorization Act (TVPRA) of 2008, unaccompanied minors from countries other than Mexico or Canada cannot be sent back to their countries of origin without a court hearing (Collier, 2015). This law states that unaccompanied minors must be held humanely by the Department of Health and Human Services (HHS) until the Office of Refugee Resettlement (ORR) releases them to a sponsor or family found suitable to care for the child(ren) (Avila, 2014; Collier, 2015; Pierce, 2015). Since March 1, 2003, ORR has been responsible for over 175,000 URM, following requirements set forth by the Flores Agreement in 1997, the Trafficking Victims Protection Act of 2000, and the TVPRA of 2005 and 2008 (Office of Refugee Resettlement, n.d.).

Following apprehension, URM must be turned over to HHS and placed in US detention centers and shelters within 72 h (Collier, 2015; Hennessy-Fiske, 2015). Once placed, URM must wait at least 21 days after being charged as unauthorized to see an immigration judge for their first hearing (Pierce, 2015). In the meantime, URM wait in detention centers, often in deplorable conditions. Despite the law’s stipulation for humane treatment, these detention centers or “hieleras,” Spanish for iceboxes or freezers (Cantor, 2015), are often extremely cold, overcrowded, with limited access to restrooms, medical care, food and water (Cantor, 2015; Collier, 2015). These conditions serve as pervasive reminders that the threat of perceived risk to survival is still present. This notion is supported by Gudiño (2013), positing that children with sensitivity to cues that threaten their survival may experience reinforcement that danger is still present when surrounded by a hostile environment after resettlement.

Detention centers and shelters have also posed other threats to URM. It has been reported that URM have been sexually and physically abused by detentions’ facility staff during their temporary placements (Collier, 2015). Brané (2018) adds that “perpetrators have included local police and ICE employees, as well as contract guards and fellow detainees.” It is evident that these statistics are difficult to monitor as these incidents often go unreported. The Department of Justice (DOJ) declared detention centers exempt from the Prison Rape Elimination Act (PREA) (ACLU, n.d.). The PREA protects people in custody from sexual abuse by setting standards for prevention, detection, and reporting (ACLU, n.d.). Without PREA enforcement, there are no laws in place to protect URM in detention centers from human rights violations and further increasing vulnerability and exposure to post migration trauma.

#### Community Integration

While awaiting court proceedings, URM are released to a relative, sponsor, or suitable families usually residing in large Central American communities in the US (Collier, 2015; Hennessy-Fiske, 2015; Pierce, 2015). After sponsors and guardians are fingerprinted and vetted by FBI and Homeland Security, they will be verified to not pose a risk to the child’s well-being (Hennessy-Fiske, 2015). Home studies are required by ORR only in cases where the URM have been reported to have a disability, history of abuse, or human trafficking. However, the homes of many sponsors and/or guardians without criminal histories, still may not be appropriate placements for these youth. Therefore, when family members or sponsors are unavailable, URM are placed in foster care (Crea, Lopez, Taylor, & Underwood, 2017). Scholars (Crea et al., 2017; Pine & Drachman, 2005) note that URM enter the child welfare system for reasons pertaining to migration and resettlement such as migratory status and exposure to traumatic events during the pre-migration and in-journey phases. In other cases, URM have reportedly experienced having food being withheld by relatives, sexual abuse, and being forced to work rather than attend school. ORR has expressed not having jurisdiction once a child is placed and that it is up to local child protective agencies to respond to cases of suspected abuse and neglect (Hennessy-Fiske, 2015). These circumstances often warrant the support of school social workers to aid in the protection of URM after placement with parents, guardians, and in some cases, with foster parents. It is evident that once placed, URM may continue being vulnerable to a host of acute and complex stressors.

Other post-migration stressors such as acculturative stress (Cano et al., 2015) and low socio-economic status (SES) contribute to emotional distress for URM. After resettlement, the SES of the family or new living arrangement (Smid et al., 2011; Yearwood et al., 2007) may be a stressor that continues to exacerbate pre-migration trauma pertaining to poverty, limited opportunities to engage in childhood activities such as play, and having to take on adult responsibilities (Martinez, 2009). URM who arrive between the ages of thirteen and seventeen often come to the US unaccompanied by a parent and face the choice of going to secondary school or directly to work (Allard, 2015). Family circumstances often pressure students to make financial contributions by forcing them to seek jobs after school, in turn resulting in challenges related to studying at home and completing homework (Abrego & Gonzalez, 2010). Other research concurs that URM arrive to the United States with adult responsibilities such as working and paying bills (Allard, 2015; Martinez, 2009). Consequently, school attendance for most migrant children is dictated by the financial needs of the family and those needs may change from day to day depending on the general economic condition (Green, 2003).

URM under the age of eighteen must make difficult decisions between working or going to school and, because of pre-migration factors, consider themselves adults with no other alternative (Allard, 2015; Martinez, 2009). Similar literature (Lukes, 2014) purports that interrupted schooling in Mexican and Central American youth is less related to a disinterest in school, but rather because of economic reasons pertaining to pre- and post-migration stressors. Educators and health providers often do not understand the complex nature that underlies the balancing act between the URM’s academic and work life. The reasons for these absences may not always be evident to educators, therefore placing the onus of school attendance on the parents and/or guardians of URM. State mandated reporters find themselves in ethical dilemmas when they question whether or not the teen is being neglected or improperly supervised, as schools label this as truancy or educational neglect resulting in the involvement of the child welfare system. Additionally, because of fear and lack of trust in authority figures, URM will fail to state the reason for the absences, unless they have established a trusting rapport with someone at the school.

Additional barriers such as anti-immigration policies in the US contribute to acute stress after resettlement for URM. Effective September 2017, the program known as Deferred Action for Childhood Arrivals (DACA) program which protected many undocumented youth from being deported, was terminated (Sessions, 2017). In addition, a decision to terminate the Temporary Protected Status (TPS) for migrants from El Salvador increased anxiety among URM residing with parents or guardians who had been granted this protection (Nielsen, 2018). The dismantling of these programs and protections result in the disruption of family structures as a result of deportation (Planas & Carro, 2017). The fear of deportation and public safety act as ongoing barriers and stressors in the lives of URM in the US.

## The Role of Trauma on Learning and Academic Achievement

Fischer (2010) posits that Mexican and Guatemalan children living in US immigrant communities are vulnerable to experiencing challenges in the educational system. The literature (Free, Križ, & Konecnik, 2014; Green, 2003, p. 65) reports that the consequences of hardships such as poverty, depression, and lack of parental presence in URM and voluntary immigrant children may result in challenges pertaining to educational mobility and exacerbate issues of illiteracy. Additional research (Collier, 2015; Dods, 2015; Pumariega et al., 2005) indicates that trauma related symptomatology may manifest in concentration, learning problems, and academic functioning for URM. Additionally, URM, who are particularly at risk among migrant groups, have reported higher levels of conduct disorders and school-related aggressive behaviors, due to prior exposure to war and violence (Pumariega et al., 2005). In this regard, exposure to trauma can impact “all areas of a youth’s life” (Dods, 2015, p. 113). This is further supported by Dods’ (2015) qualitative case study in which personal interviews were administered to three youths to explore the intersection of trauma, school experiences, and their perceptions of the roles schools play. Dods (2015) further emphasized that trauma triggers, such as a loud, crowded classroom can contribute to an increase in a student’s arousal levels. Thus, the student’s behaviors may appear oppositional and unpredictable coupled with inattention, memory, and motivation challenges. The research concluded that “unmet needs and diminished sense of agency” were common themes across participants (Dods, 2015, p. 129). The participants in Dods’ study (2015) reported that while in school, they felt guarded and unsafe. When heightened, these feelings may lead to pervasive PTSD symptoms (Smid et al., 2011) and ongoing disruptions in academic achievement.

## The Necessity for School-Based Interventions

There were approximately 840,000 immigrant students in the US in grades k-12 (Adelman & Taylor, 2015). Fortunately, schools have been identified as the primary provider of mental health services for all youth, including serving as the first social and institutional space that URM encounter (Bal & Perzigian, 2013; Beehler et al., 2012; Kataoka et al., 2003; Sullivan & Simonson, 2016). Additionally, schools have been noted to be in unique positions in providing URM school-based mental health services, thereby contributing to the reduction in emotional distress and academic challenges (Dods, 2015). Most students, k-12, spend at least 7 hours a day in school. This places school administration and staff *in loco parentis*, or in place of a parent, of students with diverse academic, social-emotional, linguistic, and cultural needs.

As with instruction, mental health services are not a one-size-fits-all model. Therefore, schools are challenged to self-assess to best respond to the complex needs of URM and voluntary immigrant students (Bal & Perzigian, 2013). As URM enter the U.S. with past or current histories of PTSD, an increase in awareness about the presence of school-based mental health services has emerged amongst educators and policy makers to promote best academic and social-emotional outcomes (Bal & Perzigian, 2013; Dods, 2015; Nadeem, Jaycox, Kataoka, Langley, & Stein, 2011). Although some literature (Beehler et al., 2012; Stein et al., 2002) posits that few studies have explored effectiveness of school-based mental health programs, schools with accessible mental health services have been effective in demonstrating improvement in students’ academic achievement, social-emotional functioning, and behavior (Nadeem et al., 2011; Unterhitzenberger et al., 2015).

School-based mental health services provide other benefits for URM, voluntary immigrant students, and their families. For example, most URM and voluntary immigrant youths need psychological treatment, however are not receiving it for a variety of reasons that may include stigma, finances, or lack of health insurance (Beehler et al., 2012; Polk, Page, & Ross DeCamp, 2014; Pumariega et al., 2005). Families of URM must often prioritize obtaining basic needs over addressing mental health needs (Beehler et al.,  2012; Murray et al., 2008). In addition to accessibility, school-based interventions help reduce culturally assigned stigma to receiving treatment (Pumariega et al., 2005; Sullivan & Simonson, 2016, p. 508). Since cultural and financial factors can be accounted for in a school setting, school-based interventions may assist in alleviating some of the obstacles that hinder URM from receiving treatment (Sullivan & Simonson, 2016). Thus, schools are situated to shorten the gap in service delivery for families who would not otherwise have access to these services (Stein et al., 2002; Sullivan & Simonson, 2016).

### Trauma-Informed and Culturally Responsive Interventions in Schools

For schools to successfully engage URM in school-based mental health services, these interventions are more likely to be effective when they are evidence-based, trauma-informed, and culturally responsive. Evidenced-based, trauma informed treatment can effectively address the unique mental health issues manifested by URM, especially when delivered by a culturally responsive provider (Beehler et al., 2012). Although, “few programs have been rigorously evaluated” (Kataoka et al., 2003, p. 311) specifically for URM and other children belonging to minority groups, the few programs that have been investigated have proven success in trauma symptom reduction and academic improvement (Nadeem et al., 2011; Unterhitzenberger et al., 2015). Additionally, Unterhitzenberger et al. (2015) posit that no study has explicitly investigated PTSD treatment in URM (p. 2). Notwithstanding, there are many trauma-informed modalities that target the reduction of trauma related symptomatology in children and adolescents. However, the literature (Bal & Perzigian, 2013; Beehler et al., 2012; Kataoka et al., 2003; Murray et al., 2008; Nadeem et al., 2011; Stein et al., 2002; Sullivan & Simonson, 2016; Unterhitzenberger et al., 2015) purports that Cognitive Behavioral Therapy (CBT), Trauma-Focused Cognitive Behavioral Therapy (TF-CBT), Cognitive Behavioral Intervention for Trauma in Schools (CBITS)—including CBITS in Spanish, and the Mental Health for Immigrants Program (MHIP) have been evaluated to be most effective in the treatment in URM and other immigrant minors in schools.

CBT is a therapeutic modality developed by Aaron T. Beck that uses attention to thoughts, behaviors, and feelings associated with thought distortions and catastrophic thinking (Beehler et al., 2012; Murray et al., 2008; Sullivan & Simonson, 2016). CBT and adapted CBT techniques and models currently “have the most empiric evidence” (Cohen, Mannarino, Berlinger, & Deblinger, 2000; Murray et al., 2008, p. 588) in treating traumatized youth. Despite the challenges of time limitations and URM’s potential low writing, reading or drawing proficiencies, CBT and adapted CBT models have also proved to be positive interventions for war traumatized refugee populations (Murray et al., 2008). TF-CBT, CBITS, and MHIP are among the most common and effective school-based interventions that use CBT techniques to reduce PTSD, depression, and anxiety symptoms in URM.

TF-CBT was developed using CBT techniques for the treatment of children with psychological sequelae of sexual abuse and has expanded to treat other forms of trauma (Beehler et al., 2012, p. 159). This manualized framework is an intensive, short-term, and time-limited approach which usually takes place over 12–16 weeks, once a week, in 60–90 min sessions (Beehler et al., 2012; Cohen et al., 2000; Sullivan & Simonson, 2016; Unterhitzenberger et al., 2015). TF-CBT emphasizes the use of exposure, cognitive processing and reframing, stress management, parental treatment, and cross-cultural modification while maintaining fidelity (Cohen et al., 2000, p. 1202; Sullivan & Simonson, 2016; Unterhitzenberger et al., 2015, p. 2). The acronym PRACTICE (P. Psychoeducation and parenting skills, R. Relaxation, A. Affective modulation, C. Cognitive processing, T. Trauma narrative, I. In vivo desensitization, C. Conjoint child/parent sessions, E. Enhancing safety and future skills) best describes each of the components used in TF-CBT (Murray et al., 2008; Unterhitzenberger et al., 2015).

Unterhitzenberger et al. (2015), purport that TF-CBT is “the best supported therapy for traumatized young people at the moment …with proven feasibility for refugee children” (p. 2). Unterhitzenberger et al. (2015), investigated the feasibility of TF-CBT in a sample of traumatized URM. The results showed a “clinically significant symptom reduction at posttest for all cases receiving TF-CBT” (p. 7). For this reason, CBT and TF-CBT “may be useful in school systems serving large populations of refugees” (Sullivan & Simonson, 2016, p. 523). Overall, TF-CBT has been proven to reduce PTSD, internalizing, and externalizing symptoms in traumatized youth (Murray et al., 2008). Furthermore, CBT and TF-CBT are feasible interventions that can be used by school social workers, guidance counselors, school psychologists, and other educational and behavioral specialists in treating children exposed to violence and complex trauma (Kataoka et al., 2003; Sullivan & Simonson, 2016, p. 523.)

Like TF-CBT, CBITS is a skills-based group intervention, developed by using a community-partnered research model, that uses cognitive behavioral techniques in relieving depression, anxiety, and PTSD symptoms in trauma exposed children, ages 10–15 (NCTSN, 2012; Nadeem et al., 2011). It was initially developed to effectively decrease trauma related symptomatology for ethnically diverse and immigrant youth in schools (Ngo et al., 2008). Interventions are delivered in 10, 1-h group sessions composed of 6–8 children, once a week. The protocol includes one to three individual sessions, two parent sessions, and one teacher education session (NCTSN, 2012) and teaches participants six cognitive-behavioral techniques: (1) Education about reaction to trauma, (2) Relaxation training, (3) Cognitive therapy, (4) Real life exposure, 5., Stress or trauma exposure, and 6. Social problem-solving (NCTSN, 2012). To account for cultural diversity, CBITS programs are available in Spanish and there are efforts being made in adapting CBITS for Native American children (NCTSN, 2012).

To address the gap in effective evidenced-based, trauma-informed interventions in schools, in 1998 a team of clinician researchers from the RAND corporation, the University of California, Los Angeles (UCLA), and the Los Angeles Unified School District (LAUSD) collaborated in developing effective interventions (Nadeem et al., 2011; Stein et al., 2011). The school partners in the development of CBITS wanted to offer a program that could effectively be delivered by school staff, in 45-min class periods, to a large number of students in a linguistically and culturally responsive way (Nadeem et al., 2011). In their study, Stein et al. (2011) demonstrated that participants exposed to violence who received CBITS experienced significant improvement, evidenced by decrease in PTSD symptoms. In support of this literature, a randomized controlled study by Ngo et al. (2008) established a significant decrease in depressive and PTSD symptoms in Mexican and Central American youth (p. 858). The implementation of CBITS in schools has broadened nationally (California, Colorado, Louisiana, New Mexico, and New Jersey) and internationally (Australia, China, Japan, and Guyana) (Stein et al., 2011). As a result, CBITS is a recommended, effective school-based intervention in treating URM, immigrant youth, and other ethnically and linguistically diverse youth in effectively decreasing trauma related symptoms by “integrating cultural-sensitivity and evidenced-based practice” (Ngo et al., 2008, p. 861; Stein et al., 2011).

MHIP is a school-based mental health intervention that incorporates CBT and is based on the CBITS framework (Kataoka et al., 2003; Stein et al., 2002). In the development of MHIP, LAUSD Mental Health Services Unit (MHSU) developed this framework by considering the school and cultural ecologies, to thereby effectively address the needs of Los Angeles’ large Latino immigrant student body (Kataoka et al., 2003; Stein et al., 2002). Hence, program materials were made available in Spanish and other languages (Stein et al., 2002). In addition to CBT, MHIP also included home visits to meet with parents or guardians and were school-based to minimize barriers to service access (Stein et al., 2002). Services were provided during times when students were both in school and the school was able to provide adequate space in order to avoid decline in participation (Stein et al., 2002). Overall, MHIP aimed at reducing PTSD symptoms for immigrants who had been exposed to violence, sexual abuse, and other traumas (Kataoka et al., 2003).

The study conducted by Kataoka et al. (2003) demonstrated that MHIP was associated with a modest reduction in symptoms, however, further research is needed to determine sustainability and replication by other schools. Stein et al. (2002) add that although many challenges in school-based mental health research exists, MHIP provided a useful framework to guide program development in the future (p. 324). Stein et al. (2002) purport that future program engagement with recent immigrant parents or guardians of migrant children would be an asset to MHIP’s development. In addition to being trauma-informed, school-based interventions are increasingly effective when culturally responsive modifications are made (Isakson, Legerski, & Layne, 2015).

### Challenges with Implementation in Schools

CBT, TF-CBT, CBITS and MHIP are all models that effectively target the reduction of trauma symptoms in URM and immigrant youth. However, there are some challenges in implementing these models in schools with this population. As stated earlier, URM migrate to the U.S. unaccompanied by a parent or guardian. In many cases, parents of URM migrated to the U.S. when the child(dren) was or were very young. Sometimes, URM leave their parents in their countries of origin and join grandparents, aunts, uncles, or other family members whom they have never met. Since all of these models include a parent psychoeducational piece, it is important to note that the absence of collaborating with a parent or guardian who knows the URM well may present a challenge in treatment. Smith, Lalonde, and Johnson (2004) note that staggered patterns of migration, or serial migration, when parents migrate to a new country years before their children, results in stress for the family and in negative effects on the self-esteem and behavior of URM. Although there is no available literature on the effect of staggered migration on treatment in schools, it is possible that these strained family dynamics may require treatment other than those mentioned in this article.

CBT, TF-CBT, and CBITS are time limited with regard to duration of each session as well as the treatment model. These time constraints may pose challenges in schools regarding student schedules and potential inconsistent attendance of URM. As a result, treatment may be interrupted or subject to sudden programming changes by the school.

The aforementioned treatment modalities, also rely on writing, drawing, and/or reading. In some cases, URM might enter the U.S. school system with interrupted education in their countries of origin, varying levels of cognitive development, and/or limited writing and reading skills. School social workers may adapt the modalities by using a verbal or dramatic form of expression (Murray et al., 2008).

### Cultural Responsiveness: Considerations in School-Based, Trauma-Informed Treatment

Although schools have been identified as the front-line providers of mental health services to minors, including URM and voluntary immigrant youth (Kataoka et al., 2003; Stein et al., 2002; Sullivan & Simonson, 2016), most URM’s treatment needs are not being met in schools (Sullivan & Simonson, 2016). Often linguistic and cultural barriers, such as stigma about the concept of therapy itself, may pose obstacles in identifying URM and families in need (Murray et al., 2008; Sullivan & Simonson, 2016). School staff often misinterpret trauma-triggered behaviors in URM since these may range from irritable outbursts to internalized symptoms that are less noticeable (Cohen, Mannarino, & Murray, 2011; Sullivan & Simonson, 2016). Some of these symptoms will manifest in a variety of ways including somatic complaints such as headaches and stomachaches (Cohen et al., 2011; Isakson et al., 2015; Ngo et al., 2008; Pumariega et al., 2005; Sullivan & Simonson, 2016). In support of this literature, Isakson et al. (2015), assert that “non-Western cultures do not differentiate between physical and emotional health” (p. 4). Therefore, it is important to identify how migration trauma presents in URM. Since culture shapes emotional vocabulary and meaning associated with trauma, necessary steps must be taken so that the school social worker is speaking the same cultural language as the child (Isakson et al., 2015, p. 4; Murray et al., 2008, p. 593). By doing so, school social workers are able to provide a culturally informed understanding of the URM’s behavior to the rest of the school community.

Culturally responsive interventions and approaches that validate and incorporate the cultural values, norms, and resilience factors of URM, have resulted in increased receptiveness to treatment when compared to models without cultural modifications (Fong, 2007; Isakson et al., 2015). Cultural modifications include targeting the unique needs of refugees such as housing, language, emotional and physical health (Isakson et al., 2015). For instance, CBT, TF-CBT, CBITS, and MHIP school-based interventions have been adapted to working with URM by making linguistic modifications and incorporating customs and rituals from the children’s native culture (Ngo et al., 2008; Stein et al., 2002). Although Spanish is the most commonly spoken language in Mexico and Central America, Garifuna and Mayan languages such as K’iche’ and Mam are spoken by over 500,000 people in these regions (Carcamo, 2016). Some countries in Central American use *vos*, the second person singular pronoun, instead of the informal command *tú* when speaking to family members and close friends (Alvarenga, 2017). As a result, Spanish-speaking service providers may need to familiarize themselves with variations of spoken Spanish and other languages in the treatment of URM.

Other modifications can be evidenced by incorporating cultural values and norms when meeting with parents, guardians, or family members of URM, such as leveraging the voice and input from the mother, being mindful of the role of spirituality in treatment, and being mindful of somatic complaints in a cultural context. For these reasons, it is important for school social workers and other school-based mental health staff to familiarize themselves with the culture of each URM’s country of origin.

The Cultural Adjustment and Trauma Services (CATS), is a school-based mental health model that uses CBT and TF-CBT with immigrant youth in two New Jersey school districts (Beehler et al., 2012). In this model, CBT techniques were used “eclectically as needed in response to clinical presentation” (Beehler et al., 2012, p. 159). The manualized intervention used, TF-CBT, was discontinued in the CATS model because some students found it challenging to focus on a single trauma narrative or stressor, making the implementation counterproductive. As previously mentioned, migration trauma is a tripartite process which may be composed of repeated and prolonged events that may result in complex post-traumatic stress disorder (C-PTSD). As a result, adaptable treatments that can be culturally modified to the specific experiences of URM are essential. Nonetheless, TF-CBT was used to inform other services provided by the treatment such as family involvement (Beehler et al., 2012).

Ngo et al. (2008) assert that CBITS was flexibly developed to meet the needs of ethnically and heterogeneous school districts. Additionally, this intervention was developed to strike a balance in maintaining the treatment fidelity while emphasizing cultural and systems competence. Because CBITS is not comprised of culturally specific core components, clinicians modified psychoeducation, cognitive-restructuring, and problem-solving, in working with URM who had crossed the US-Mexico border, so that the model would be sensitive to migration trauma (Ngo et al., 2008, p. 860). Operating under the notion of cultural responsiveness, CBITS involves necessary steps to not disregard URM’s family beliefs such as ghosts or other cultural beliefs (Ngo et al., 2008, p. 860). Stein et al. (2002), emphasize that like CBITS, MHIP providers collaborated in ways to address cultural issues that emerged in sessions such as belief in ghosts or spirits of deceased family members.

The models CBT, TF-CBT, CBITS, and MHIP shared additional culturally responsive modifications. All models emphasized the use of bicultural and bilingual licensed clinicians to provide services (Beehler et al., 2012; Stein et al., 2002). The literature (Beehler et al., 2012; Murray et al., 2008; Ngo et al., 2008; Pumariega et al., 2005; Stein et al., 2002) asserts that the use of *cultural brokers* or liaisons to the community with cultural knowledge and clinical expertise is crucial in assisting with outreach and in ensuring that interventions were implemented in a culturally sensitive way.

The following case example illustrates a cultural adaptation of TF-CBT in the treatment of PTSD with a URM in an urban public high school. Implementation of a manualized intervention such as TF-CBT and CBITS was not possible at this school setting due to challenges around student programming and physical space. As a result, this trauma-informed group was an adaptation of TF-CBT techniques and utilized a culturally responsive approach, including group facilitation in Spanish. This group focused on trust building, psychoeducation, trauma exposure, and consultation with parents.

### Case Example: Yaretzi

Yaretzi is a 16-year-old girl in her sophomore year at a small, New York City public high school. She is originally from a low-income neighborhood in a small town in El Salvador. Yaretzi currently lives with her paternal grandmother, a relative whom she never met or lived with before. Living with her grandmother and her strict rules is one of the many adjustments that pose as stressors for Yaretzi. She arrived in the United States, through the US-Mexico border assisted by a coyote, as an unaccompanied refugee minor. In El Salvador, Yaretzi lived with her mother, father, and two younger siblings. She reported witnessing a lot of gang violence and often felt unsafe. She remembered that “while aboard the bus to school, witnessing a group of gang members board the bus, shoot, and kill two fellow classmates.” The gang threatened the passengers, stating that if the authorities were notified there would be retribution. Yaretzi stated that her “life changed after witnessing the murder of the two students,” who allegedly had been gang affiliated. Her father drove her to school for the next couple of months, but her experience of ongoing fear, nightmares and flashbacks of the event ultimately interrupted her ability to perform daily tasks, including concentrating on school work. Despite her parents’ requests, Yaretzi dropped out of school for a year and stayed home out of fear for her safety. Yaretzi expressed that her parents wanted her to “have the opportunity of acquiring an education and a better life.” However, she did not know that her parents, in collaboration with family members living in New York City, collected money to finance her journey to the U.S. Yaretzi states that she doesn’t remember much of the journey, except that she cried the entire time and was often told to “shut up” by the coyote. After 11 days, Yaretzi arrived at the U.S.–Mexico border from El Salvador. She was apprehended in Texas and taken to a detention center.

About 2 months after her admission to the high school, Yarezti’s teachers noted to the school social worker that she had been steadily losing weight, missing school days, and falling asleep in class. One of her teachers facilitated an introduction to the school social worker. The school social worker introduced the support services offered to URM at the high school, including group and individual sessions in Spanish. Yaretzi appeared hesitant to disclose personal information to the teacher and the school social worker in the introductory conversation. However, Yaretzi made an appointment to speak to the school social worker the next day. Yaretzi was introduced by one of her peers (and group members) to the school-based counseling group designed to address the needs of URM.

### Assessment and Interventions: Trauma-Informed Treatment with Cultural Modifications

In the initial individual session (and in subsequent sessions), the school social worker met with Yaretzi in Spanish. Early in the session, Yaretzi stated concerns about confidentiality. The social worker explained the limits of confidentiality as a school official. Yaretzi reported to the social worker that she felt sad on a daily basis, experienced nightmares and interrupted sleep, and flashbacks of the event on the bus in El Salvador. She stated having difficulty focusing in class, primarily because much of the content was taught in English. Although she had made a couple of friends, Yaretzi reported feeling that she “did not belong” here. Yaretzi also stated that she “hated living with her grandmother because she was strict and mean.” She added that food in the U.S. tasted “like plastic, artificial. Nothing like mom’s tortillas back at home and other tasty food.” The school social worker discussed the migration process with Yaretzi. Yaretzi spend some time discussing the negative and positive aspects of her life in El Salvador, the hardships endured in her journey to the U.S., and the stressors she had been facing after her arrival to New York City. In the initial session, the school social worker assessed for depression and self-harm. At the conclusion of the session, the school social worker invited her to join the group. Yaretzi stated that she would think about it.

Yaretzi and one of her peers approached the school social worker’s office the next day requesting to join. The group consisted of five Central American high school girls, ages 15–16, met once a week for 40 min, throughout the 10-month academic year. As suggested by the literature (Cohen et al., 2011), when youth may have been exposed to multiple traumas, as observed in migration’s tripartite process, TF-CBT’s *Enhancing Safety* skill of the PRACTICE model may be introduced before *Psychoeducation*. Focusing on this skill, the social worker was able to develop an environment of safety in a group of girls that had faced threatening events in their native countries and in their respective journeys to the U.S. Safety and trust-building was created through group activities that encouraged reliance on each other. During this process, Yaretzi was often hesitant to participate, but after encouragement from peers, she joined activities more readily. Yarezti and other group members identified family, church, priests, *Santeros, Espiritistas, Curanderas*, and other practitioners of traditional medicine as sources of safety in their communities.

The school social worker introduced *psychoeducation* about PTSD, depression, and anxiety symptoms related to the migration process and slowly normalizing exposure to trauma. Psychoeducational sessions were also held with parents and/or guardians of the group members. These sessions took place in person or by phone in order to adjust to the families’ work schedules. Engaging the family in discussions pertaining to the group’s purpose, migration trauma, trauma symptoms and stigma helped parents and guardians feel like stakeholders in the students’ (and their own) healing process. Yaretzi’s grandmother was grateful for the existence of the group on her granddaughter’s life, however, she was mostly concerned with her academic success and following the rules at home. Cultural understandings around trust, shame, discipline, respect, hierarchy, and family dynamics are the important values to be aware of in conversations with families and in treatment with URM.

When the group was ready, they began to explore and share their *trauma narrative***—**the exposure to trauma related memories through narrative. This was done through journal entries, visual art, songs, acting and story-telling. For example, the group members engaged in creative ways to help construct the narrative such as: *“What happened the day before you left (country of origin)?”, “What event(s) stands out about your journey to the U.S.?” and “What do you remember about the exact moment you arrived in the U.S.?”* Yaretzi wrote a poem and a story depicting her experiences before, during, and after migration. The trauma narratives developed and changed over a few weeks while emerging feelings and memories were processed in group. In her narrative, Yaretzi emphasized a longing for “the beauty of her country,” her mother’s cooking, the smell of her home in El Salvador, the sadness she felt about being “forced to leave,” and “living in a strange country.” In the post-migration narrative, Yaretzi expressed feeling “stressed having to balance the role between being a student and having to work full time after school.” After a month of participating in the group and seeing the school social worker for individual sessions, Yaretzi’s effort in class improved, her attendance became more consistent, and she stopped sleeping in class.

## Discussion

This case example illustrates the necessity for exploring migration trauma as a tripartite process including the provision of school-based, trauma-informed interventions. During pre-migration, Yaretzi witnessed the death of peers and was threatened by the gang members that murdered the youngsters on the school-bound bus. Fearing for her safety after the shooting, Yaretzi’s education in El Salvador was interrupted, sleep patterns were dysregulated by nightmares, and capacity to focus on daily tasks became increasingly challenging. It is important to note that prior to the shooting, Yaretzi was exposed to poverty and other stressors that accompany low-income families in the Northern Triangle. As noted throughout the literature, stressors that accompany poverty may contribute to PTSD up to 9 years prior to migration (Fong, 2007; Rasmussen et al., 2012; Smid et al., 2011; Yearwood et al., 2007).

In keeping with this notion, the literature suggests that exposure to gang violence and witnessing the violent death of someone are pre-migration stressors that increase susceptibility to PTSD (Fortuna et al., 2008; Rasmussen et al., 2012; Smid et al., 2011; Yearwood et al., 2007). In Yarezti’s case, she was exposed to these stressors from birth. Ultimately, these interruptions and acute stressors acted as push factors that pressured Yaretzi’s family to plan her migration to the US. Moreover, these events forced Yaretzi out of El Salvador without appropriately saying goodbye to many loved ones in addition to leaving cherished possessions, memories, and components of her culture behind.

Once embarked, Yaretzi did not witness or experience violence or physical risks. However, throughout the migration journey, Yaretzi experienced loss, separation, grief, profound sadness, and distress. She was met with no support while being forced to stifle the expression of emotions by the coyote. Vargas (2014) explains that “*coyotes* are well known in Central American townships, and the smuggling business has traditionally been built on trust. Knowing the right coyote is key to securing a safe journey” (para.7). Although Yaretzi did not undergo other forms of abuse by the coyote, she reported feeling “demeaned” by the coyote’s demands to stop crying. Yaretzi’s trauma narrative as depicted in her story and poem, highlight the anguish she experienced from the sudden loss of family, friends, and community. Thus, traumatic grief over the unexpected separation from her family and birthplace may have added to the complexity of the pre-existing trauma experienced by Yaretzi (Murray et al., 2008).

Yaretzi’s resettlement and adjustment experiences after migration also contributed to pre-existing stressors. Post-migration stressors that may have exacerbated her feelings of loss, danger, and grief were the low socio-economic status in the new living arrangement with her grandmother and recurrent nightmares and flashbacks about the shooting. The literature explains that after re-settlement, poverty in the new country may act as a continuous stressor while negatively impacting educational outcomes in URM (Collier, 2015; Dods, 2015; Free et al., 2014; Green, 2003; Pumariega et al., 2005). Consequently, Yaretzi reported appetite loss, change in eating habits, and decrease in energy. As a result, academic achievement, body weight, and capacity to concentrate in and out of school were directly impacted. Teacher referrals and established supports such as the school-based, trauma-informed student group, individual sessions with the school social worker in Spanish, and establishing peer supports allowed for timely identification of possible PTSD symptoms and adjustment challenges. Since schools are considered to be at the frontline in mental health service provision for minors, Yaretzi was able to develop a safe, support system in a trusted space. Most importantly, support was delivered in Yaretzi’s native language and her narrative was understood from a culturally responsive, trauma-informed perspective.

## Practice, Policy, and Research Implications

### Practice and Policy Implications

Although it has been noted that URM have unique needs that call for tailored interventions, most are not receiving the necessary mental health services within schools or in their respective communities. The literature reviewed emphasized the complexity in the treatment of immigrant youth, in particular, Mexican and Central American URM. Voluntary immigrant youth and URM have reported differences in PTSD prevalence. URM have been identified as a vulnerable and high-risk group susceptible for developing PTSD and other psychological sequelae as a result of exposure to stressors throughout the migration process. There is a danger in pathologizing symptoms and behaviors in URM if clinicians in settings that serve URMs, especially in schools, do not assess for trauma as discussed in this article. Knowledge of URMs socio-political and economic histories, values, norms, and resilience factors would enhance the delivery of culturally responsive treatment.

Therefore, an in-depth exploration of migration’s tri-partite process as it pertains to trauma and other psychological sequelae may assist mental health providers in school deliver culturally responsive services to these youth. Additionally, schools would better support URM by offering social work services in the student’s native language wherever possible. School staff should make active efforts in understanding the norms and values of the student’s culture and incorporating these into trauma-informed services. The support of the student’s parents, guardians, family members, and cultural brokers in the community should be leveraged as part of the treatment process in schools.

Overall, it is proposed that school-based, mental-health service providers and educators become attuned to the unique needs of URM. Therefore, culturally adjusting trauma-informed interventions to the complex events experienced by URM throughout the migration process (Isakson et al., 2015; Murray et al., 2008). Neglecting this connection results in learning and adjustment difficulties that may exacerbate pre-existing PTSD symptoms.

### Implications for Research

Because of current gaps in research, longitudinal, multimodal, and participatory action research (Stein et al., 2002) may help bridge research disparities for the purpose of translating findings into practice. Further research is needed pertaining to the provision of culturally responsive, trauma informed treatment of URM in schools. Moreover, the literature consistently reports that studies about effective school-based, trauma treatment of Mexican and Central American URM, are scarce (Beehler et al., 2012; Dods, 2015; Isakson et al., 2015; Murray et al., 2008). This dearth in the literature may be partly due to challenges associated with studying URM as a population (Collier, 2015). For instance, fear of immigration status disclosure and cultural beliefs and stigma about mental health services may contribute to these challenges (Collier, 2015; Murray et al., 2008; Sullivan & Simonson, 2016). Other challenges are posed by concerns pertaining to translating evidence-based treatment into practice and balancing model fidelity with culturally responsive modifications (Kazdin, 2008; Ngo et al., 2008). While CBT and TF-CBT have been reported to be feasible and effective school-based interventions, other literature states that TF-CBT is less effective in URM with complex trauma as noted throughout the tripartite process of migration (Beehler et al., 2012; Sullivan & Simonson, 2016).

## Conclusion

This article explored migration as a traumatic, tripartite process as it relates to the unique needs of URM and school-based services (Fazel & Stein, 2002; Kirmayer et al., 2011; Perez-Foster, 2001; Sullivan & Simonson, 2016). The influx of URM in the US continues to rise steadily and may continue to do so in years to come (Pierce, 2015; Yearwood et al., 2007). URM are reportedly at a higher risk for developing PTSD and other psychological sequelae as a result of their migration process when compared to voluntary migrants. For this reason, schools are in unique positions to provide mental health services to URM that are culturally-responsive and trauma informed. CBT, TF-CBT, CBITS, and MHIP have been effective school-based models in the treatment of URM, although some literature argues that TF-CBT is less effective in youth with complex trauma. The effectiveness of these models was enhanced by adopting linguistic modifications to the model’s protocols, delivering services in Spanish by bilingual/bicultural social workers, and incorporating the youth’s cultural norms and values wherever possible.

However, literature pertaining to the treatment of URM in schools is scarce. It is essential that school districts nationwide re-evaluate the services being provided to students in English as a New Language (ENL) classes (formerly English as a Second Language) which is where most URM are placed upon admission to US schools. It is important that school district leaders look beyond the students’ academic needs to include social-emotional needs in the form of trauma-informed supports. Thus, training must be provided for school social workers, other school-based mental health staff, teachers, guidance counselors, and administration.
